# Saccharomyces genome database update: server architecture, pan-genome nomenclature, and external resources

**DOI:** 10.1093/genetics/iyac191

**Published:** 2023-01-04

**Authors:** Edith D Wong, Stuart R Miyasato, Suzi Aleksander, Kalpana Karra, Robert S Nash, Marek S Skrzypek, Shuai Weng, Stacia R Engel, J Michael Cherry

**Affiliations:** Department of Genetics, Stanford University, Stanford, CA 94305, USA; Department of Genetics, Stanford University, Stanford, CA 94305, USA; Department of Genetics, Stanford University, Stanford, CA 94305, USA; Department of Genetics, Stanford University, Stanford, CA 94305, USA; Department of Genetics, Stanford University, Stanford, CA 94305, USA; Department of Genetics, Stanford University, Stanford, CA 94305, USA; Department of Genetics, Stanford University, Stanford, CA 94305, USA; Department of Genetics, Stanford University, Stanford, CA 94305, USA; Department of Genetics, Stanford University, Stanford, CA 94305, USA

**Keywords:** *Saccharomyces cerevisiae*, budding yeast, SGD, model organism database, knowledgebase, MOD

## Abstract

As one of the first model organism knowledgebases, *Saccharomyces* Genome Database (SGD) has been supporting the scientific research community since 1993. As technologies and research evolve, so does SGD: from updates in software architecture, to curation of novel data types, to incorporation of data from, and collaboration with, other knowledgebases. We are continuing to make steps toward providing the community with an *S. cerevisiae* pan-genome. Here, we describe software upgrades, a new nomenclature system for genes not found in the reference strain, and additions to gene pages. With these improvements, we aim to remain a leading resource for students, researchers, and the broader scientific community.

## Introduction

The *Saccharomyces* Genome Database (SGD; www.yeastgenome.org) is a knowledgebase with a purpose to collect, organize, and annotate scientific data about the yeast *Saccharomyces cerevisiae*. A knowledgebase is an online resource that integrates, curates, and organizes published results into an online resource that provides easy access and reusability of these information. SGD's content is managed by a group of dedicated, PhD-trained scientists who screen published research articles, manually curate the information, and connect it to other relevant resources. The curation team works closely with our expert software developers to create a robust website that presents yeast genetic and cellular biology research in a manner that is useful for both experimental and computational scientists. In recent years, software technologies have advanced to provide services and data more quickly. To streamline SGD's framework, we continue to update our website architecture to make use of cloud services and move away from managing physical servers toward serverless compute engines.

Since its creation, SGD has been the authority for the *S. cerevisiae* reference genome (http://sgd-archive.yeastgenome.org/sequence/S288C_reference/; Cherry *et al.* 2012), including gene annotation and nomenclature (Cherry 1995). We have recently updated the reference genome annotation, expanded our collection of allele information, and are developing the Alliance of Genome Resources in partnership with five other model organism knowledgebases and the Gene Ontology (GO) Consortium (Engel *et al.* 2022). Although S288C is the strain from which the yeast reference genome sequence is derived, many publications use other strain backgrounds that contain genes not found in S288C (Engel *et al.* 2016; Song *et al.* 2016). To accommodate these genes so that we can fully serve and support the yeast research community, we are moving toward providing an *S. cerevisiae* pan-genome. We have recently established a new systematic nomenclature for these genes not found in S288C, which we describe below.

Outreach to the community and working with researchers to incorporate published data is another important part of SGD's work (MacPherson *et al.* 2017). In recent years, we have collaborated with the ComplexPortal (Meldal *et al.* 2022) to curate and to incorporate macromolecular complex data into SGD, resulting in new complex pages covering the complete yeast complexome (Wong *et al.* 2019). We have also worked directly with researchers to incorporate genome-wide protein abundance data into SGD protein pages (Nash *et al.* 2020). We continue to work with the community to incorporate new connections to external databases, including the recent addition of RNA secondary structures, described below.

## Updating server architecture

**Fig. 1.**
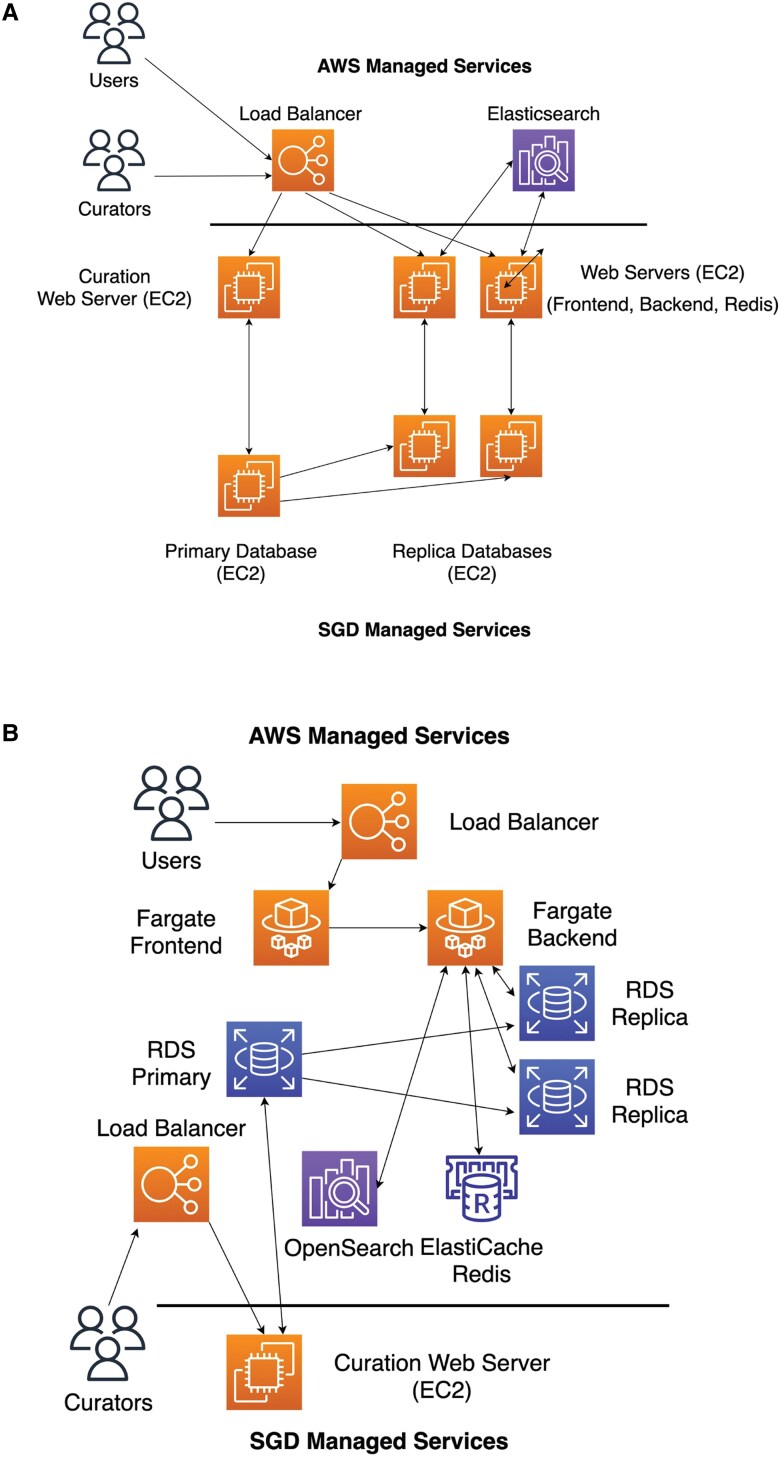
Streamlining SGD's backend with Fargate and Docker. AWS managed services are above the horizontal line, SGD managed services are below. a) Previous architecture of SGD. AWS managed services are limited to load balancer and elasticsearch; all other services are SGD managed. b) Current architecture, with SGD managed services reduced to the curation web server.

As new web and cloud technologies develop, we evaluate which would be most appropriate for SGD and of most benefit to our users. SGD has migrated our server infrastructure to utilize Docker containers (https://www.docker.com/resources/what-container/) running under the Amazon Web Services (AWS) Fargate (https://aws.amazon.com/fargate/) service (Fig. 1). Docker is a platform that enables developers to build, deploy, and run software as immutable “containers,” which are uniform, executable components that comprise the entire application runtime environment including code, libraries, and dependencies. Because each container is “complete,” the software can be written a single time and, like a virtual computer, can run anywhere like desktops, servers, and the cloud, which reduces the need for time-consuming refactoring. A Docker application can therefore be deployed with greater speed, security, reliability, and flexibility than traditional software deployments. As an example, SGD utilizes the same Docker image across all four of our major computing environments: development, QA, staging, and production. Deployment to any environment can be performed with just a few clicks from a management console or by running a simple command-line script. SGD also shares our Docker images to any interested user via the Amazon Elastic Container Registry (ECR, https://aws.amazon.com/ecr/) Public Repository (https://gallery.ecr.aws/yeastgenome/). If users wish to customize their own Docker image, SGD includes a modifiable Dockerfile as part of our source code repositories located at https://github.com/yeastgenome/.

AWS Fargate is a “serverless” environment, managed and operated by AWS. By using Fargate, SGD is no longer responsible for server management tasks that are necessary to maintain normal server operations, but which do not add direct value to the website end user. Performing data backups, upgrading operating system software, and managing security patches are responsibilities now handled by AWS (Fig. 1b). By transferring this nondifferentiated work to AWS, SGD staff can direct attention away from systems management and instead focus on adding data and features that directly benefit our end users. This increased efficiency adds value and resiliency to the site, which ultimately reduces costs for SGD in the long run as well.

Fargate detects when a container has become unresponsive and will automatically replace that container with a newly launched one. Fargate also implements auto-scaling, meaning it will launch new containers to handle spikes in demand as needed, as well as deactivate containers when the additional compute capacity is no longer needed. By leveraging such “elasticity,” SGD optimizes cost-effectiveness by deploying only the amount of compute resources that are needed to handle user demand at any given moment.

Initial SGD deployment of Docker containers in Fargate has resulted in modest cost savings. SGD expects these savings to increase over time as more applications are migrated from Elastic Compute Cloud (EC2) instances to Fargate, as SGD realizes increased economy of scale due to differences in data storage models. In the future, SGD also expects to maximize cost-efficiency by migrating some Docker containers to Lambda (https://aws.amazon.com/lambda/), the AWS serverless, event-driven service that charges only for compute and memory resources actually used when a job is invoked. Unlike EC2 and Fargate, Lambda charges do not accrue while awaiting the arrival of user requests.

## Toward a pan-genome: new systematic names for genes not found in S288C

A number of *S. cerevisiae* genes are found in alternative background strains, but not in the reference genome of S288C. As these features are not in the reference genome sequence, systematic chromosomal locations are not defined. Historically, SGD has cataloged these genes using only their gene names. As gene names can change and are sometimes ambiguous, we have put into place a new systematic nomenclature that accommodates all genes in the yeast pan-genome that are not found in S288C. This new systematic nomenclature is similar to, but distinct from, those used for ORFs (Cherry 1995; Cherry *et al.* 1998) and for ncRNAs (Engel *et al.* 2022). This new nomenclature is for those genes not found in S288C. For those genes that are found in S288C, the established systematic nomenclature remains the same. New genes that are identified in both S288C and non-S288C strains will also be given systematic gene names following the current, established systematic nomenclature when the reference genome is updated (Engel *et al.* 2022).

Nonreference genes are designated by a symbol consisting of three uppercase letters and a four-digit number, as follows: Y for “Yeast,” SC for “*Saccharomyces cerevisiae*,” and a four-digit number corresponding to the sequential order in which the gene was added to SGD. We currently have 55 of these genes in SGD, some of which are old favorites like maltose permease (MAL21/YSC0004) and the mating-type locus (MATA/YSC0046), while others are more recent additions like xylitol dehydrogenase (XDH1/YSC0051; Table 1). Going forward, as researchers publish evidence pointing to other *S. cerevisiae* genes not present in the S288C reference genome, they will be added to the annotation using the next sequential number available. Already, we have an additional 15 of these YSC names reserved by researchers and awaiting publication.

**Table 1.** New systematic gene names for genes not found in the reference genome.

Systematic name	Standard gene name	Gene name description	Gene description
YSC0001	ENS2	ENdo.SceI	Mitochondrially encoded 50 kDa subunit of Endo.SceI
YSC0002	KHR1	Killer of heat resistant	Killer toxin
YSC0003	MAL2	MALtose fermentation	Multigene complex, polymeric locus for maltose fermentation
YSC0004	MAL21	MALtose fermentation	Maltose permease
YSC0005	MAL22	MALtose fermentation	Maltase (alpha-D-glucosidase)
YSC0006	MAL23	MALtose fermentation	MAL-activator protein
YSC0007	MAL34	MALtose fermentation	ORF with sequence and positional similarity to the MAL64 gene
YSC0008	MAL4	MALtose fermentation	Multigene complex, polymeric locus for maltose fermentation
YSC0009	MAL41	MALtose fermentation	Maltose permease
YSC0010	MAL42	MALtose fermentation	Maltase (alpha-D-glucosidase)
YSC0011	MAL43	MALtose fermentation	MAL-activator protein
YSC0012	MAL6	MALtose fermentation	Multigene complex, polymeric locus for maltose fermentation
YSC0013	MAL61	MALtose fermentation	High-affinity maltose transporter
YSC0014	MAL62	MALtose fermentation	Maltase (alpha-D-glucosidase)
YSC0015	MAL63	MALtose fermentation	MAL-activator protein
YSC0016	MAL64	MALtose fermentation	MAL64 is a nonfunctional homolog of the MAL63 trans-activator
YSC0017	MATA1	MATing type	Expressed copy (at MATa) of a1
YSC0018	MATA2	MATing type	Protein of unknown function
YSC0019	MEL1	MELibiose	Secreted alpha-galactosidase
YSC0020	MEL10	MELibiose	Secreted alpha-galactosidase
YSC0021	MEL2	MELibiose	Secreted alpha-galactosidase
YSC0022	MEL3	MELibiose	Secreted alpha-galactosidase
YSC0023	MEL4	MELibiose	Secreted alpha-galactosidase
YSC0024	MEL5	MELibiose	Secreted alpha-galactosidase
YSC0025	MEL6	MELibiose	Secreted alpha-galactosidase
YSC0026	MEL7	MELibiose	Secreted alpha-galactosidase
YSC0027	MEL8	MELibiose	Secreted alpha-galactosidase
YSC0028	MEL9	MELibiose	Secreted alpha-galactosidase
YSC0029	MPR1	sigMa 1278b gene for L-proline analog resistance	L-azetidine-2-carboxylic acid acetyltransferase
YSC0030	NTS1-1	Nontranscribed spacer	Nontranscribed region of the rDNA repeat between the 3′-ETS and RDN5
YSC0031	RF2	Reading frame 2	Maturase-like coding sequence downstream of COX3/Q0275
YSC0032	RTM1	Resistance to toxic molasses	Member of the lipid-translocating exporter family
YSC0033	STA1	STArch	Glucoamylase (glucan 1,4-alpha-glucosidase)
YSC0034	STA2	STArch	Glucoamylase (glucan 1,4-alpha-glucosidase)
YSC0035	STA3	STArch	Starch hydrolysis
YSC0036	STRP	*S. cerevisiae* TM30nm related protein	Transmembrane protein with a highly basic C-terminal region
YSC0037	SUC1	SUCrose	Invertase
YSC0038	SUC3	SUCrose	Invertase
YSC0039	SUC4	SUCrose	Invertase
YSC0040	SUC5	SUCrose	Invertase
YSC0041	SUC7	SUCrose	Invertase
YSC0042	AWA1	AWA (Japanese for “foam”)	Putative GPI-anchored protein
YSC0043	BIO6	BIOtin	Putative 7-keto-8-aminopelargonic acid (KAPA) synthetase
YSC0044	KHS1	Killer of heat sensitive	Thermolabile killer toxin encoded on the right arm of Chromosome V
YSC0045	TAT3	Tyrosine amino acid transporter	Permease identified in lager brewing yeast strain Weihenstephan Nr.34
YSC0046	MATA	MATing type	Mating-type locus
YSC0047	BIO1	BIOtin	Putative pimeloyl-CoA synthetase involved in biotin biosynthesis
YSC0048	BIO8	BIOtin	Putative KAPA synthetase
YSC0049	BIO7	BIOtin	Putative pimeloyl-CoA synthetase involved in biotin biosynthesis
YSC0050	ENA6	Exitus NAtru (Latin, “exit sodium”)	Plasma membrane sodium-pumping ATPase
YSC0051	XDH1	Xylitol deydrogenase	Xylitol dehydrogenase
YSC0052	SUC8	SUCrose	Invertase
YSC0053	FPG1	Foam-promoting gene	Putative cell wall mannoprotein involved in foam formation
YSC0054	IMI1	Irr1-mediated interaction	Protein involved in maintaining mitochondrial integrity and glutathione homeostasis
YSC0055	MPR2	sigMa1278b gene for L-proline analog resistance	L-azetidine-2-carboxylic acid acetyltransferase

## Additional visuals and external links: structure and homology

**Fig. 2.**
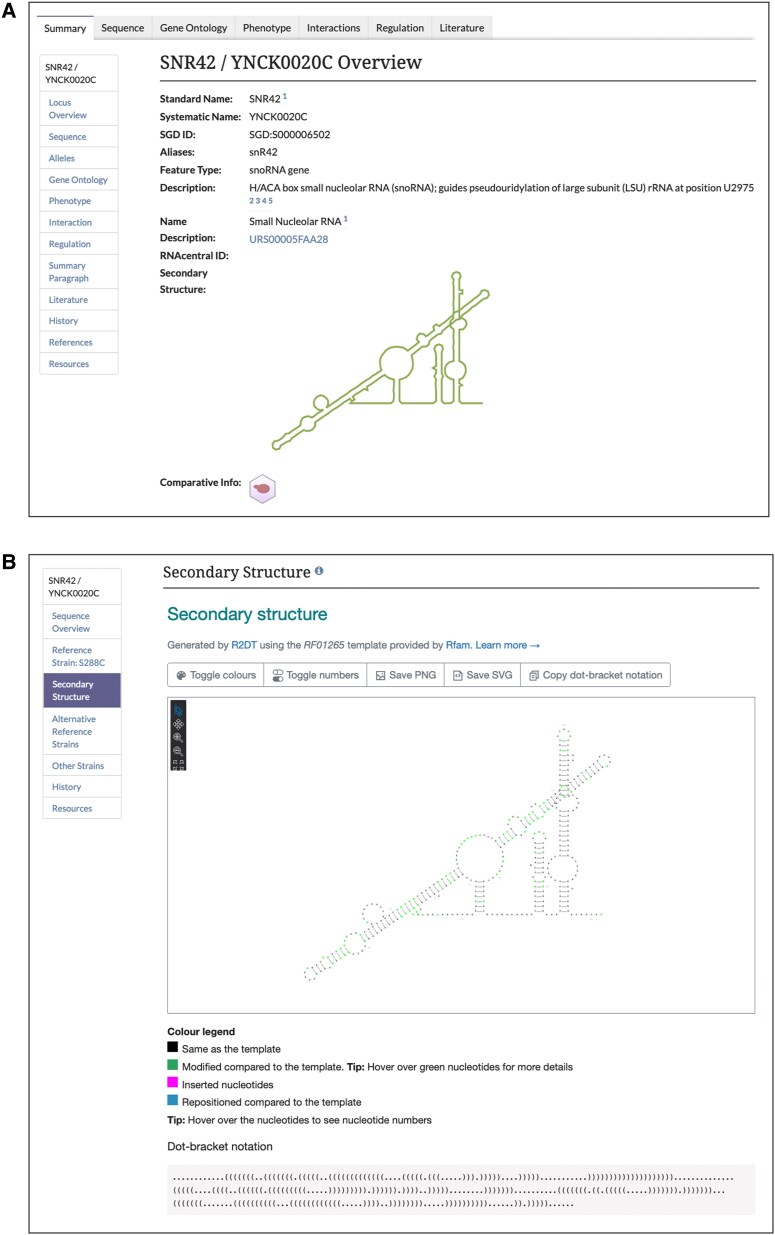
Secondary structures from RNAcentral are now available on SGD RNA gene pages. a) Thumbnail view of SNR42/YNCK0020C secondary structure on Locus Summary page and (b) detailed view of secondary structure of SNR42/YNCK0020C on Sequence page.

SGD continues to incorporate relevant information from external databases (Wong *et al.* 2019; Nash *et al.* 2020). We have recently added secondary structures for RNAs from RNAcentral (https://rnacentral.org/; RNAcentral Consortium 2021). Secondary structure images can be found on both the Summary and Sequence pages for RNA genes (Fig. 2a). The Sequence pages have a more detailed view of the secondary structures (Fig. 2b). Additionally, RNAcentral IDs are provided as links directly to the corresponding RNAcentral page for more information.

Another way to connect users to external structural and homology databases is through the Resources section at the bottom of the Gene and Complex pages. Within the Resource section of the Summary, Sequence, Protein, and Homology pages, we have added links to the corresponding entry at the AlphaFold Protein Structure Database (https://alphafold.ebi.ac.uk/). Similarly, in the Resource section on Complex pages, we added links to ModelArchive (https://www.modelarchive.org/), a database of predicted 3-dimensional structures of macromolecular complexes. A newly developed database, AnalogYeast (https://www.weizmann.ac.il/molgen/AnalogYeast/), predicts homologous proteins by sequence similarity in other organisms, including humans and other model organisms such as *Caenorhabditis elegans* and *Arabidopsis thaliana* (Cohen *et al.* 2022). We added links to AnalogYeast's search results within the Resource sections of the Protein and Homology pages to enable researchers to explore homologs of disease-associated genes.

## Continuing directions

As SGD enters its fourth decade, we continue to evolve our database, website architecture, and user interface, keeping it sustainable and current to ensure the website and all curated data are easily accessible for both users and computers. As we progress toward the *S. cerevisiae* pan-genome, equipped with the new systematic nomenclature, we have the infrastructure to easily add new, published gene features that are not found in the S288C reference sequence. As founding members of the GO Consortium (http://geneontology.org/; Ashburner *et al.* 2000; Gene Ontology Consortium 2021) and the Alliance of Genome Resources (https://www.alliancegenome.org/; Alliance of Genome Resources Consortium 2022), as well as being the authority for *S. cerevisiae* for GenBank, NCBI Gene, UniProtKB, and RNACentral, SGD has a long history of partnering with other databases. SGD will continue to actively participate in these collaborations, including working both with other databases and directly with researchers, to continue to connect fundamental yeast research with other research communities. We validate the accuracy of the external links as they are added to the site, as well as when an error is reported. Additionally, we encourage and welcome community feedback through our helpdesk, sgd-helpdesk@lists.stanford.edu, on any enhancements or new features that we make.

## Data Availability

All information and materials provided by SGD are available with the Creative Commons Attribution 4.0 International (CC BY 4.0) license. This license allows others to distribute, remix, adapt, and build upon the information or materials, even commercially, as long as credit to the source is provided.
